# Dissemination of Quinolone-Resistant Escherichia coli in the Norwegian Broiler and Pig Production Chains and Possible Persistence in the Broiler Production Environment

**DOI:** 10.1128/AEM.02769-19

**Published:** 2020-03-18

**Authors:** Håkon Kaspersen, Camilla Sekse, Eve Zeyl Fiskebeck, Jannice Schau Slettemeås, Roger Simm, Madelaine Norström, Anne Margrete Urdahl, Karin Lagesen

**Affiliations:** a Norwegian Veterinary Institute, Oslo, Norway; b Institute of Oral Biology, Faculty of Dentistry, University of Oslo, Oslo, Norway; University of Naples Federico II

**Keywords:** QREC, AMR, quinolone, livestock, wildlife, *Escherichia coli*, animals, antimicrobial resistance, genomics

## Abstract

In Norway, the use of quinolones in livestock populations is very low, and prophylactic use is prohibited. Despite this, quinolone-resistant Escherichia coli (QREC) isolates are present at low levels in several animal species. The source of these QREC isolates is unknown. The aim of this study was to characterize and compare QREC isolates from different animal species to identify putative factors that may promote the occurrence of QREC. A total of 280 QREC isolates, from broilers, pigs, red foxes, and wild birds, were whole-genome sequenced and analyzed. Well-known chromosomal and plasmid-mediated resistance mechanisms were identified. In addition, mutations in *marR*, *marA*, and *rpoB* causing novel amino acid substitutions in their respective proteins were detected. Phylogenetic analyses were used to determine the relationships between the isolates. Quinolone resistance mechanism patterns appeared to follow sequence type groups. Similar QREC isolates with similar resistance mechanism patterns were detected from the samples, and further phylogenetic analysis indicated close evolutionary relationships between specific isolates from different sources. This suggests the dissemination of highly similar QREC isolates between animal species and also the persistence of QREC strains within the broiler production chain. This highlights the importance of both control measures at the top of the production chain as well as biosecurity measures to avoid the further dissemination and persistence of QREC in these environments.

**IMPORTANCE** Since antimicrobial usage is low in Norwegian animal husbandry, Norway is an ideal country to study antimicrobial resistance in the absence of selective pressure from antimicrobial usage. In particular, the usage of quinolones is very low, which makes it possible to investigate the spread and development of quinolone resistance in natural environments. Comparison of quinolone-resistant E. coli (QREC) isolates from livestock and wild animals in light of this low quinolone usage provides new insights into the development and dissemination of QREC in both natural and production environments. With this information, preventive measures may be taken to prevent further dissemination within Norwegian livestock and between other animals, thus maintaining the favorable situation in Norway.

## Abstract (precis)

Since antimicrobial usage is low in Norwegian animal husbandry, Norway is an ideal country to study antimicrobial resistance in the absence of selective pressure from antimicrobial usage. In particular, the usage of quinolones is very low, which makes it possible to investigate the spread and development of quinolone resistance in natural environments. Comparison of quinolone-resistant E. coli (QREC) isolates from livestock and wild animals in light of this low quinolone usage provides new insights into the development and dissemination of QREC in both natural and production environments. With this information, preventive measures may be taken to prevent further dissemination within Norwegian livestock and between other animals, thus maintaining the favorable situation in Norway.

## INTRODUCTION

Quinolones are broad-spectrum antimicrobial compounds that have been used to treat infections in both humans and animals all over the world and are included in the highest-priority group on the WHO’s list of critically important drugs for human medicine. Unfortunately, the extensive use of quinolones has resulted in the emergence of quinolone-resistant bacteria. As part of a combined effort to manage the increasing problem of antimicrobial resistance, national and international surveillance programs have been established to monitor the occurrence and spread of resistant bacteria, including quinolone-resistant Escherichia coli (QREC), in livestock animals (1, 2). The overall occurrences of quinolone resistance among commensal E. coli isolates from broilers and fattening pigs in Europe in 2016 and 2017 were 64.0% and 10.6%, respectively, although the occurrence varies considerably between countries (1, 3). These values were based on the epidemiological cutoff (ECOFF) values for ciprofloxacin defined by the European Committee on Antimicrobial Susceptibility Testing (EUCAST) (www.eucast.org). Similar resistance levels were reported for nalidixic acid. To our knowledge, no systematic surveillance has been done on wild animals at the European level.

Since 2000, the Norwegian monitoring program for antimicrobial resistance in feed, food, and animals (NORM-VET) has monitored antimicrobial resistance in commensal E. coli isolates from a range of animal species (5). In NORM-VET, antimicrobial susceptibility to a panel of substances, including quinolones, is determined by testing the susceptibility of randomly selected isolates using broth microdilution (5, 46). In addition, a directly selective method for detecting QREC in samples from animals was introduced in 2014 (4). In Norway, the use of fluoroquinolones in livestock populations is very low (5), and prophylactic use is prohibited. This is reflected in a low occurrence of quinolone resistance among commensal E. coli isolates as documented through NORM-VET reports. For example, the overall occurrence of quinolone resistance among commensal E. coli isolates from broilers, pigs, red foxes, and wild birds from 2006 to 2017 was 1.8%, ranging from 0.3% in pigs, 1.24% in red foxes, and 2.3% in wild birds to 2.9% in broiler flocks (data retrieved from the NORM-VET database). QREC has nevertheless been detected with the selective method in a high proportion of samples from these animal species (4, 6, 7). The overall occurrence of QREC detected by selective screening performed in the years 2014 to 2017 among the above-mentioned animal species was 37.1%, ranging from 14.8% in red foxes, 20.4% in wild birds, and 54.4% in pigs to 79.2% in broilers (boot swab samples from broiler production breeder flocks were included in 2017). Although the number of positive samples from broilers seems higher than that from pigs, it has to be taken into account that broiler samples are pooled samples of 10 animals per flock, while pig samples are from individual animals representing the pig herd.

The broiler production system in Norway has a pyramidal structure with high levels of biosecurity. Grandparent eggs are imported from Scotland to Sweden before hatching. Eggs from these grandparent animals are then imported to Norway to become parent animals, whose day-old chickens are distributed to broiler farms across the country. In contrast, pig production in Norway is a purely domestic system with a negligible import of live animals. Although pig production also has a pyramidal structure, it has considerably more movement of animals between farms.

Quinolone resistance mechanisms in E. coli have been thoroughly characterized and are for the most part mediated by chromosomal mutations in the quinolone resistance-determining regions (QRDRs) of *gyrA*, *gyrB*, *parC*, and/or *parE* (8). Mutations in several other chromosomal regulatory genes (e.g., *marA*, *soxRS*, and *robA*) or mutations in *rpoB* (RNA polymerase B) have also been implicated (9–12). Additionally, plasmid-mediated quinolone resistance (PMQR), such as the *qnr* family of genes, *qepA*, *oqxAB*, and *aac(6′)-Ib-cr*, has been described (13–16).

The aim of the present study was to compare QREC isolates originating from four different animal species (broilers, pigs, red foxes, and wild birds), tested for susceptibility within the framework of NORM-VET from 2006 to 2017. For these purposes, whole-genome sequencing of the isolates and subsequent analyses were performed. The relationships between isolates were analyzed by phylogenetic approaches with the intent to elucidate possible dissemination within and between animal species. In addition, genetic characterization of quinolone resistance and plasmid-mediated resistance to other antimicrobials was performed.

## RESULTS

### Quinolone resistance gene identification.

**(i) Chromosomal genes.** Mutations resulting in amino acid substitutions were detected in seven of the nine chromosomal genes investigated. In total, 229 of the 280 isolates had substitutions in the QRDR of GyrA, 43 isolates had substitutions in ParC, and 29 isolates had substitutions in ParE (Table 1). No mutations giving rise to substitutions in the QRDR of GyrB were detected. Six different substitutions were identified in GyrA and ParC, while seven were identified in ParE (see Table S4 in the supplemental material). Isolates from broilers had the highest occurrence of substitutions in GyrA and ParE, while isolates from wild birds had the highest occurrence of substitutions in ParC (Table 1). The most frequent substitutions in the respective proteins were S83L in GyrA, S80I in ParC, and D475E in ParE (Table S4). The S83L substitution in GyrA and the D475E substitution in ParE were most often identified in isolates from broilers (Table S5), while the S80I substitution in ParC was most often identified in isolates from wild birds. A total of 231 isolates had substitutions in the QRDR of at least one of GyrA, ParC, or ParE. The most abundant combination of substitutions in the QRDRs of GyrA, ParC, and ParE was S83L in GyrA alone, found in 141 isolates. The substitutions S83L and D87N in GyrA combined with the S80I substitution in ParC occurred in a total of 33 isolates, of which 16 had only the S80I substitution, 8 had S80I combined with A56T, and 1 had S80I combined with E84V. The remaining eight isolates had the S80I substitution in ParC combined with substitutions in ParE. Regarding all three genes combined, eight isolates had substitutions in GyrA, ParC, and ParE. Considering the other chromosomal genes, 212 isolates had substitutions in MarR, 71 had substitutions in SoxR, 48 had substitutions in RpoB, and 34 had substitutions in MarA. No substitutions were identified in RobA (Table 1). The most common substitutions in each gene were S127N in MarA, G103S combined with Y137H in MarR, E320D in RpoB, and T38S combined with G74R in SoxR (Table S6). Substitutions in RpoB occurred significantly more often in isolates from broilers than in isolates from pigs [χ^2^(1,*n* = 163) = 10.95 (*P* = 0.001)] and wild birds [χ^2^(1,*n* = 153) = 5.73 (*P* = 0.017)]. Substitutions in MarA always accompanied substitutions in MarR.

**TABLE 1:** Number of isolates with mutations leading to amino acid substitutions in the included chromosomal genes and presence/absence of plasmid-mediated genes per animal species

Type of resistance	Gene	No. of isolates					% of isolates^*a*^
		Broiler (*n* = 87)	Pig (*n* = 75)	Red fox (*n* = 52)	Wild bird (*n* = 66)	Total (*n* = 280)	
Chromosomal	*gyrA*	87	56	42	44	229	81.8
	*gyrB*	0	0	0	0	0	0
	*marA*	19	2	7	6	34	12.1
	*marR*	66	52	40	54	212	75.7
	*parC*	8	9	10	16	43	15.4
	*parE*	14	5	3	7	29	10.4
	*robA*	0	0	0	0	0	0
	*rpoB*	25	6	9	8	48	17.1
	*soxR*	29	18	11	13	71	25.4

Plasmid mediated	*qepA4*	0	0	0	1	1	0.4
	*qnrA1*	0	0	1	0	1	0.4
	*qnrB19*	1	11	2	7	21	7.5
	*qnrS1*	3	6	6	14	29	10.4
	*qnrS2*	0	3	1	2	6	2.1
	*qnrS4*	0	0	1	0	1	0.4

a The percentage is relative to the total number of isolates (280).

**(ii) PMQR genes.** Plasmid-mediated quinolone resistance was identified in 59 of the 280 isolates, and only one PMQR gene type was found for each isolate (see Table 1 for the presence of PMQR-positive isolates in different animal species and the specific PMQR genes present). The occurrence of PMQR was significantly lower in isolates from broilers than in isolates from pigs [χ^2^(1,*n* = 163) = 15.78 (*P* < 0.05)], red foxes [χ^2^(1,*n* = 140) = 9.42 (*P* = 0.002)], and wild birds [χ^2^(1,*n* = 153) = 26.21 (*P* < 0.05)]. The most commonly identified PMQR genes were *qnrS1* and *qnrB19*, identified in isolates from all animal species (Table 1). Isolates from pigs had a significantly higher occurrence of *qnrB19* than isolates from broilers [χ^2^(1,*n* = 163) = 10.87 (*P* = 0.001)] and red foxes [χ^2^(1,*n* = 127) = 3.91 (*P* = 0.048)]. The occurrence of *qnrS1* was significantly higher in isolates from wild birds than in isolates from broilers [χ^2^(1,*n* = 153) = 12.44 (*P* < 0.05)] and pigs [χ^2^(1,*n* = 140) = 5.21 (*P* = 0.022)]. A strong negative correlation between the presence of *qnr* and substitutions in GyrA was observed [*r*(278) = −0.92 (*P* = 0.05)]; 49 of the 58 isolates carrying *qnr* did not have substitutions in the QRDR of either GyrA, ParC, or ParE (Table S7).

**FIG 1:**
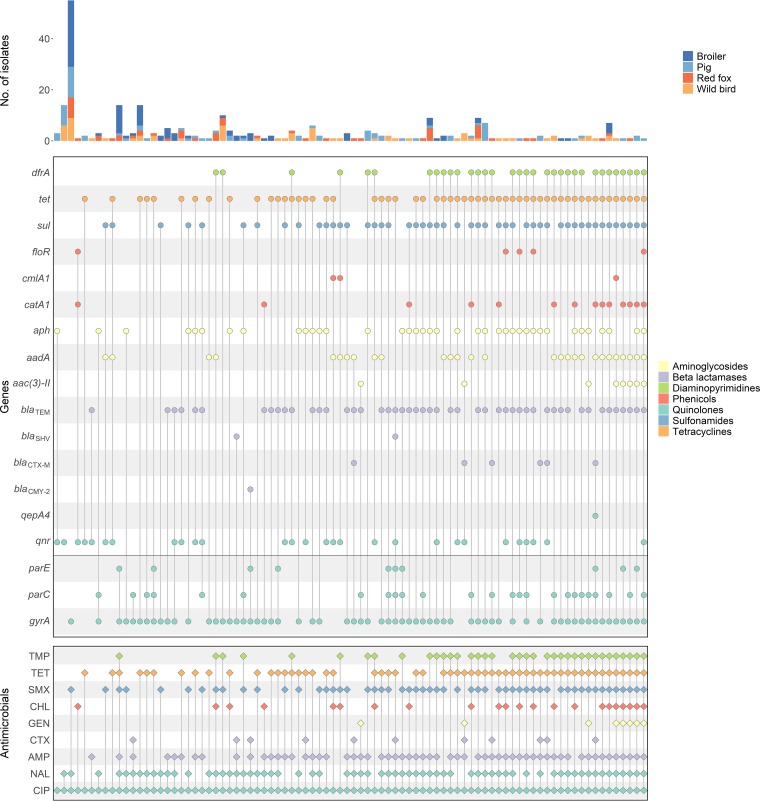
Phenotypic and genotypic resistance patterns for all plasmid-mediated resistance genes and *gyrA*, *parC*, and *parE*. The top plot represents the number of isolates per group. The middle plot represents the presence/absence of plasmid-mediated genes and chromosomal mutations (below the horizontal line). The bottom plot represents the phenotype of the respective gene/mutation combination. Meropenem and colistin were excluded as resistance was not observed among any isolates, and ceftazidime was excluded as cephalosporin resistance was already represented by cefotaxime. Tigecycline was excluded due to almost no resistance being observed among the isolates. Colors represent animal species and resistance phenotypes (TMP, trimethoprim; TET, tetracycline; SMX, sulfamethoxazole; CHL, chloramphenicol; GEN, gentamicin; CTX, cefotaxime; AMP, ampicillin; NAL, nalidixic acid; CIP, ciprofloxacin). The genes in the middle plot are grouped based on gene family [*dfrA* represents *dfrA1*, *dfrA5*, *dfrA8*, *dfrA12*, *dfrA14*, and *dfrA17*; *tet* represents *tetA*, *tetB*, and *tetD*; *sul* represents *sul1* to *sul3*; *aph* represents *aph3Ia*, *aph3Ib*, and *aph6Id*; *aadA* represents *aadA1*, *aadA2*, *aadA5*, *aadA12*, *aadA13*, and *aadA22*; *aac(3)-II* represents *aac(3)-IIa* and *aac(3)-IId*; *bla*_TEM_ represents *bla*_TEM-1A_ to *bla*_TEM-1C_; *bla*_SHV_ represents *bla*_SHV-2_ and *bla*_SHV-12_; *bla*_CTX-M_ represents *bla*_CTX-M-1_, *bla*_CTX-M-15_, *bla*_CTX-M-32_, and *bla*_CTX-M-55_; *qnr* represents *qnrA1*, *qnrB19*, *qnrS1*, *qnrS2*, and *qnrS4*].

**(iii) Coresistance.** In total, the presence of 42 different genes encoding resistance to gentamicin, cefotaxime, chloramphenicol, tetracycline, trimethoprim, and sulfamethoxazole was identified (Table S8), in addition to the PMQR genes described above. Six genes did not have a corresponding antimicrobial compound in the panel of substances against which all the isolates had previously been tested and were therefore not considered when comparing genotype to resistance phenotype. Except for a few cases, the genotype corresponded to the phenotype (Fig. 1).

In the 59 PMQR-positive isolates, *qnr* was observed as the only plasmid-mediated gene in 14 of the isolates (Table S9). Of these 14 isolates, 12 harbored *qnrB19*, and 2 harbored *qnrS2*. Among the 29 *qnrS1*-positive isolates, 22 harbored *tetA*, and 21 harbored *bla*_TEM-1B_, while among the 21 *qnrB19*-positive isolates, only 4 isolates carried *tetA*, and 6 carried both *aph3-Ib* and *aph6-Id*.

A significant positive correlation between the presence of *qnrS1* and *tetA* (0.36; *n* = 22), *dfrA14* (0.31; *n* = 8), *bla*_CTX-M-55_ (0.31; *n* = 3), *bla*_TEM-1B_ (0.26; *n* = 21), *floR* (0.22; *n* = 3), and *aac(3′)-IId* (0.12; *n* = 3) was observed (*P* < 0.05). For *qnrB19*, a significant positive correlation with *bla*_TEM-1A_ was identified (0.14; *P* < 0.05), but the two genes were observed together in only one isolate. For the 221 PMQR-negative isolates, 72 isolates had no identified plasmid-mediated resistance genes. Except for ParC, a negative correlation was observed between the presence of plasmid-mediated resistance genes and mutations in chromosomal genes (Fig. S3).

### Isolate diversity.

**FIG 2:**
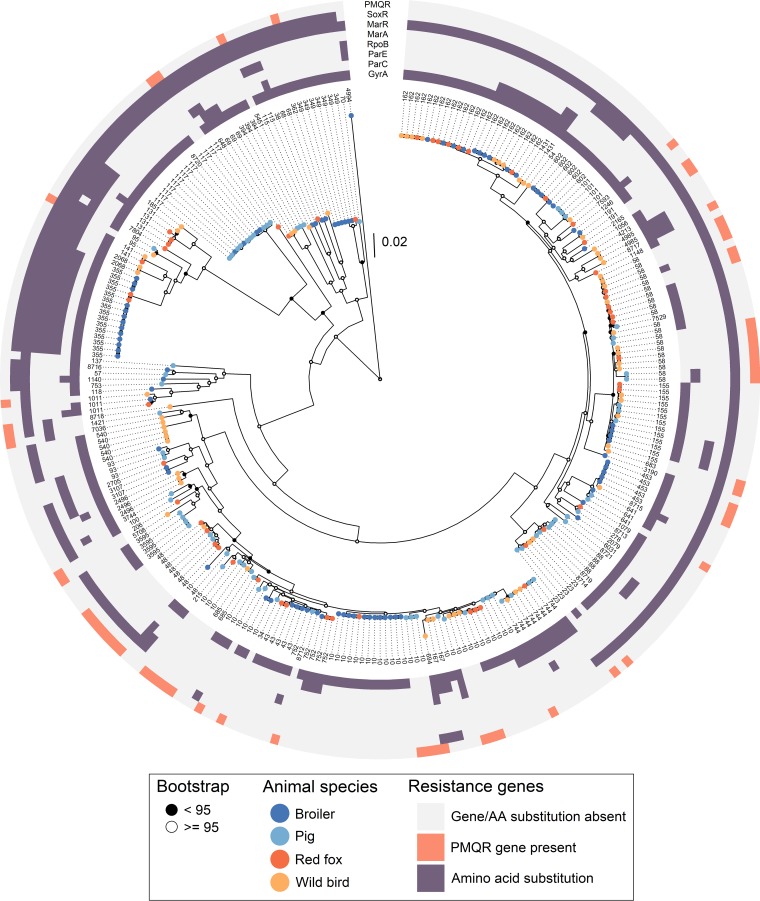
Maximum likelihood core-gene SNP tree of all isolates. Branch supports (ultrafast bootstrap approximation) are denoted with black or white nodes. The colored tips on the tree denote animal species of origin, and the tip labels denote the sequence types from the MLST scheme hosted by EnteroBase. The coloring on the outer rings denotes the presence/absence of mutations leading to amino acid (AA) substitutions in chromosomal genes and the presence/absence of plasmid-mediated genes. The tree was generated with IQTree from SNPs in core genes from Roary aligned with MAFFT. The evolutionary model used was GTR+F+ASC+R9. The tree is midpoint rooted for better visualization.

In total, 83 unique sequence types (STs) were identified, with each animal species containing between 26 and 33 different STs. The most abundant STs were ST10 (*n* = 38), ST162 (*n* = 24), ST58 (*n* = 20), ST355 (*n* = 15), ST117, and ST155 (*n* = 13). ST10 and ST155 isolates were identified in all animal species. ST162 isolates were identified in all animal species but pigs, and ST58 isolates were identified in all but broilers. ST355 isolates were identified in broilers and red foxes, while ST117 isolates were identified in broilers and pigs (Fig. 2). A total of 59 STs were present in only one animal species.

Based on the core-gene single nucleotide polymorphism (SNP) alignment, isolates from broilers had the lowest median minimum pairwise distance compared to the other animal species, indicating smaller differences between isolates from broilers than the other species (Table S10). The randomization test revealed that isolates from broilers aggregated more closely than isolates within other animal species (*P* < 0.01) (Fig. S4).

**FIG 3:**
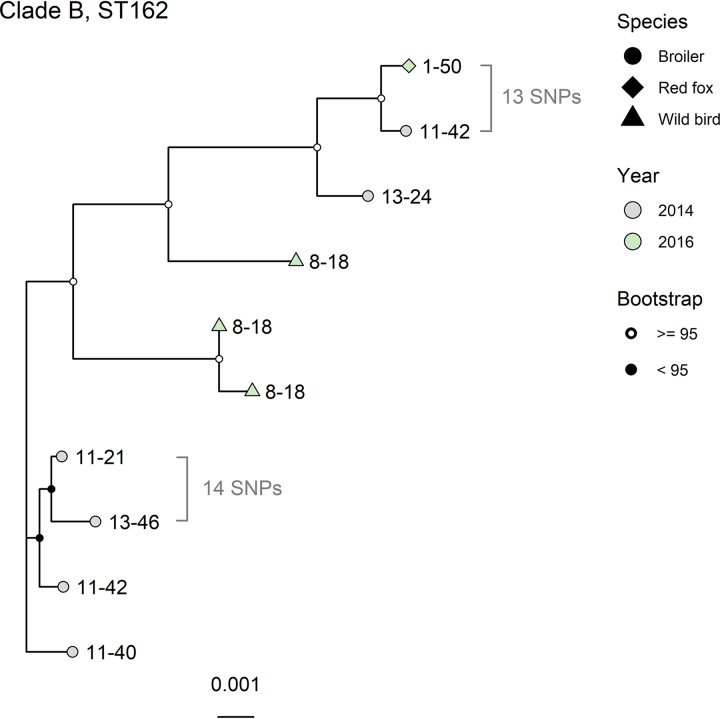
Maximum likelihood core-genome tree of clade B, containing 10 ST162 isolates. Tip labels denote the location of the isolate by county-municipality. Core-genome SNPs were called with ParSNP, recombinant sites were removed with Gubbins, and the tree was generated with IQTree. The evolutionary model used was TIMe+ASC+R2. The percentage of the genome shared among all isolates was 86%. The highly similar isolates from wild birds in this tree (location 8-18, 2016) were disregarded as they were from the same sample, one isolated by the traditional method and the other isolated by the selective method.

**FIG 4:**
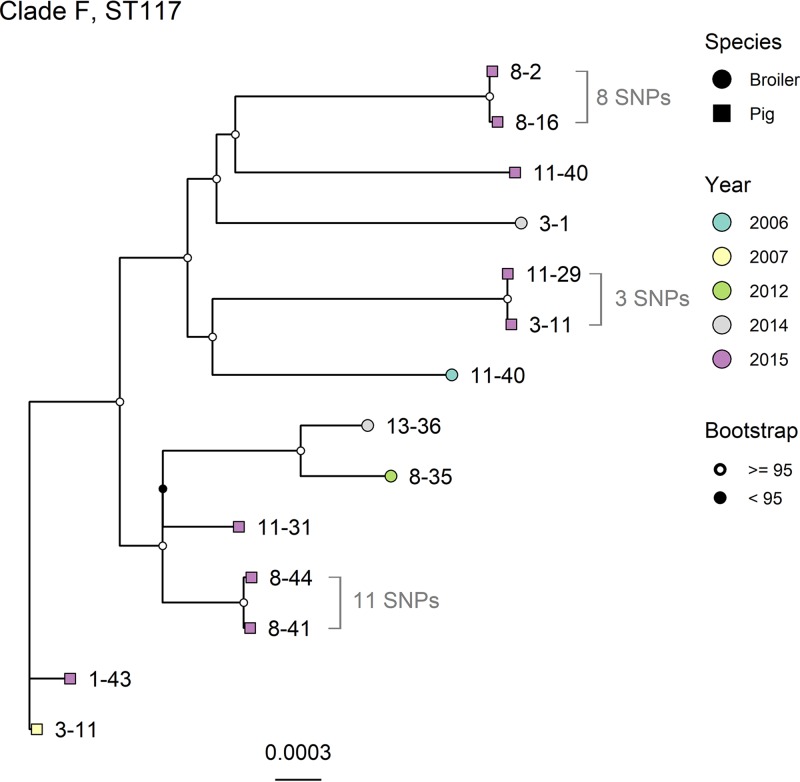
Maximum likelihood core-genome SNP tree of clade F, containing both ST117 (*n* = 13) and ST8720 (*n* = 1; from 2012) isolates. Tip labels denote the location of the isolate by county-municipality. Core-genome SNPs were called with ParSNP, recombinant sites were removed with Gubbins, and the tree was generated with IQTree. The evolutionary model used was K3P+ASC+G4. The percentage of the genome shared among all isolates was 83.6%.

Six clades were selected for deeper phylogenetic analysis, as they contained isolates with low genetic divergence and were from either different animal species or the same animal species but different geographic locations: clade A (ST162 subclade A), clade B (ST162 subclade B), clade C (ST744), clade D (ST10), clade E (ST355), and clade F (ST117) (clade selection is shown in Fig. S1). The trees for clades A, C, D, and E had low bootstrap support and were not considered further since the topology within each clade was judged to be uncertain (Fig. S5 to S8, respectively). Clade B (Fig. 3) consisted of isolates from broilers, red foxes, and wild birds, sampled in 2014 and 2016. This clade contained two pairs of isolates that were especially similar. The first pair consisted of one isolate from a broiler and one from a red fox; these had an SNP distance of 13. The host species originated from geographically distant locations and were also sampled in different years. The two isolates shared >90% of their genomes (Table 2). The second pair of isolates was from broilers in different locations in 2014. They had an SNP distance of 14 and shared >90% of their genomes. Clade F (Fig. 4) consisted of isolates from broilers and pigs, sampled in the years 2006, 2007, 2012, 2014, and 2015. All annotated isolate pairs in Fig. 4 were from pigs sampled in 2015 and had SNP distances of 8, 3, and 11 from the other isolate in the same pair. Two of these pairs shared >90% of their genomes. These two isolate pairs were from the same county but not the same municipality, while for the third pair, the isolates were from different counties. All pairs of isolates investigated had identical phenotypic and genotypic resistance patterns.

**TABLE 2:** Overview of isolates of interest from ST162 (clade B) and ST117 (clade F)^*a*^

ST	Isolate	No. of SNPs	% similarity of genomes	Source	Yr	Location
162	1	13	90.8	Red fox	2016	1-50
	2			Broiler	2014	11-42
	1	14	90.9	Broiler	2014	11-21
	2			Broiler	2014	13-46

117	1	3	95.4	Pig	2015	11-29
	2			Pig	2015	3-11
	1	8	74.1	Pig	2015	8-2
	2			Pig	2015	8-16
	1	11	91.0	Pig	2015	8-44
	2			Pig	2015	8-41

a The location identifiers represent county-municipality (anonymized). The pairs correspond to the annotated clades in Fig. 3 and 4.

**FIG 5:**
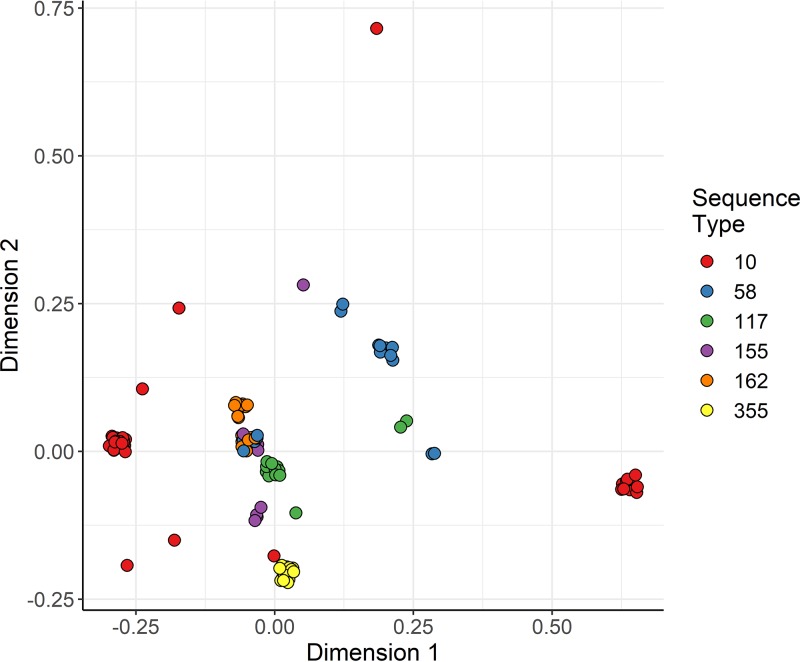
Nonmetric multidimensional scaling (NMDS) analysis of the presence/absence of quinolone resistance mechanisms, both plasmid mediated and chromosomal. The colors denote sequence types. The points are jittered for easier interpretation.

Nonmetric multidimensional scaling (NMDS) clustering of isolates based on the presence/absence of quinolone resistance mechanisms in isolates from major ST groups showed that ST355, ST155, ST117, and ST162 were relatively homogenous in their distribution of quinolone resistance mechanisms, while ST10 and ST58 were not (Fig. 5).

## DISCUSSION

This study uses whole-genome sequencing to characterize and compare a large number of QREC isolates from different animal species obtained through a monitoring program on antimicrobial resistance in animals. Although there was a high level of diversity of STs among the isolates and animal species, we show that phylogenetically similar QREC isolates were shared both between animal species and between locations. Moreover, the genetic quinolone resistance determinants found in this study predominantly clustered within STs. Taking this clustering pattern into consideration, the phylogenetic structure indicates dissemination in the broiler and pig production chains and potential persistence in the broiler production chain.

We detected some novel substitutions, one in MarR and two in MarA and RpoB, which to our knowledge have not been previously described. As it is outside the helix-turn-helix DNA binding motifs, the observed D118N substitution in MarR probably does not affect DNA binding directly (17). However, follow-up studies are needed to examine if these novel substitutions affect quinolone susceptibility. In addition, the observed cooccurrence of substitutions in MarA with substitutions in MarR and the significantly higher occurrence of substitutions in RpoB in broilers should be further investigated.

PMQR determinants were identified in 21.1% of the 280 selected isolates, with the highest occurrence of PMQR genes among the wild-bird isolates (36.7%) and with *qnrS1* being the most common determinant. The high occurrence of *qnrS* in wild birds is in concordance with previously reported data (18, 19). A positive correlation was observed between *qnrS1* and genes related to tetracycline, gentamicin, trimethoprim, chloramphenicol, ampicillin, and cefotaxime resistance. Resistance to these antimicrobials has previously been associated with *qnrS1* (20). *qnrS1* genes have previously been identified on large conjugative plasmids harboring *bla*_TEM-1B_ and *tetA* (21, 22), which supports the significant positive correlations between *qnrS1*, *bla*_TEM-1B_, and *tetA*. On the other hand, *qnrB19* genes have been found on small, nonconjugative plasmids without any other resistance genes (23). In our data, only *bla*_TEM-1A_ had a significant positive correlation with *qnrB19*, but these genes were observed together in only a single isolate. Furthermore, most *qnrB19*-positive isolates harbored no other plasmid-mediated genes. These findings may suggest that we have two main types of plasmids in our isolates, one conjugative plasmid with *qnrS1* and other resistance genes and another nonconjugative plasmid with mostly only *qnrB19*. The presence of these plasmid types appeared to cluster mainly within sequence types. However, further studies characterizing the plasmids from these isolates are needed to confirm these findings but were not performed here, as this was outside the scope of this study. The occurrence of PMQR in wild birds was noticeably higher than what has been reported in other studies (20, 24, 25). However, comparison to other studies is difficult due to differences in sampling and study design. For instance, the wild bird isolates selected in this study were not representative of the wild bird population in Norway, as sampling was performed in four regions only. These isolates therefore cannot be regarded as being epidemiologically unrelated. PMQR was detected in only four isolates from broilers. This low occurrence may be due to the high biosecurity in broiler production, with little to no contact with the outside environment. The predominance of chromosomally encoded resistance indicates that PMQR plays a minor role in the occurrence of QREC in the broiler production chain. In contrast, PMQR determinants were detected in 20 isolates from pigs, the most common one being *qnrB19*, indicating a higher occurrence of PMQR among QREC isolates in the Norwegian pig production environment. Further studies are needed to elucidate the origins of these plasmids.

An overall correspondence between genotype and phenotype was observed in our data, except for two isolates with decreased susceptibility to cefotaxime. Further investigation using PointFinder (26) identified a mutation in the *ampC* promoter region in one of these isolates (data not shown), but the decreased susceptibility remains unexplained for the other isolate. Isolates harboring *qnr* in addition to substitutions in GyrA were identified in four broiler isolates. Three of these had the same sequence type and contained *qnrS1*, indicating that the plasmids containing *qnrS1* are being clonally disseminated. In contrast, only one *qnr*-positive isolate each from pigs, red foxes, and wild birds had substitutions in GyrA. Six out of seven of these isolates showed elevated MIC values above the clinical breakpoints for ciprofloxacin (1 to 16 mg/liter) and nalidixic acid (64 to 256 mg/liter), corresponding to an additive effect of multiple quinolone resistance mechanisms. High MIC values from such an additive effect are a common finding in regard to quinolone resistance in E. coli (27, 28). Such elevated MIC values were not observed for the rest of the *qnr*-positive isolates, highlighting the need for chromosomal mutations to gain a high MIC value.

A strong negative correlation between the presence of *qnr* genes and substitutions in GyrA was observed, indicating that the two mechanisms rarely coincide. This may be explained by the hypothesized protective effect of *qnr* genes on the quinolone targets, which allows other resistance mechanisms to be developed instead of mutations in the QRDRs of these genes (29). The majority of isolates that carried *qnr* genes without substitutions in GyrA, ParC, or ParE had substitutions in MarR, which may be a consequence of this protective effect. Negative correlations were also observed for most of the investigated chromosomal genes and the plasmid-mediated resistance genes, indicating that coselection of these genes is not common in QREC isolates from animal sources in Norway. However, further studies regarding plasmid characterization and coresistance are needed to confirm these findings.

We identified a high level of diversity of STs, which has also been reported by others (20, 30, 31). Among these were STs previously associated with quinolone resistance, such as ST10, ST162, ST355, and ST349 (20, 32). Moreover, the results show that the distribution of resistance mechanisms was relatively homogenous within most STs, supporting a clonal distribution of these mechanisms. Isolates from broilers were overall more similar to each other than the isolates from the other animal species, as shown in the core-gene SNP tree and supported by the permutation test. This may be due to the centralized distribution of broilers, permitting the dissemination of QREC isolates to the entire production chain. Although there is a centralized distribution of animals in pig production as well, such an overall similarity was not observed among the QREC isolates from pigs. However, we identified two phylogenetically related pig isolates from geographically distant locations, indicating that dissemination of QREC isolates in the pig production chain may occur. Persistence of antimicrobial-resistant bacteria in broiler production environments, despite short production cycles, cleaning, and disinfection between flocks, is known from other studies (33, 34). Vertical dissemination of QREC and cephalosporin-resistant E. coli to all levels of the broiler production pyramid has previously been described for both QREC and cephalosporin-resistant E. coli (35–38) in both Norway and neighboring countries. Our results, which show close phylogenetic relationships between QREC isolates from broilers, strengthen the hypothesis that dissemination within the broiler industry originates from imported breeding animals, as suggested by Börjesson et al. (35).

Isolates from red foxes had the highest SNP distances from other isolates within the same animal species. In a previous study, Mo et al. showed that the occurrence of QREC in red foxes was low in areas with a low human population density and higher in areas with a medium or high human population density (39). Mo et al. suggested that red foxes in urban areas have been exposed to different kinds of indirect human exposures. This could contribute to the high level of diversity observed among the red fox isolates.

Interestingly, we identified phylogenetically related ST162 isolates with the same resistance mechanism patterns shared between a broiler and a red fox from geographically distant locations. One possible explanation for this is a combination of the distribution of similar isolates through the broiler production chain and that the red fox, for instance, came into contact with the isolate through broiler fecal matter used to fertilize crop fields. The two isolates in question were from different years, which may indicate the persistence of QREC in the broiler production environment. Although dissemination from red foxes to broilers cannot be ruled out, the opposite direction is more likely due to the biosecurity measures in broiler production facilities.

To summarize, this study revealed a high level of diversity in the QREC populations in the four studied animal species. Nevertheless, QREC isolates that were phylogenetically related were found both within and between host species. The phylogenetic structure also revealed that the quinolone resistance mechanisms are mostly clonal. While the origins of quinolone resistance in these populations remain unclear, these results indicate that QREC isolates in a livestock production chain may be disseminated down through the production pyramid. This highlights the importance of biosecurity-focused control measures at the top of the production chain to prevent the dissemination and persistence of QREC and PMQR in these environments.

## MATERIALS AND METHODS

### Isolate selection.

Isolates included in this study were collected in the NORM-VET program from 2006 to 2017 (4–7, 40–46). Isolate metadata can be downloaded as described in Section S3.1 in the supplemental material. In NORM-VET, the procedures for isolation were either traditional, by plating fecal, cecal, or boot swab samples on MacConkey agar (BD Biosciences, Le Pont de Claire, France), or selective, by plating samples on MacConkey agar with 0.06 mg/liter ciprofloxacin (0.12 mg/liter in 2014). For both methods, a random E. coli colony was selected from the plate and confirmed to be E. coli either by citrate, indole, and/or oxidase tests or by matrix-assisted laser desorption ionization–time of flight (MALDI-TOF) mass spectrometry (Microflex; Bruker Daltonik GmbH). The selected isolate was then tested for susceptibility by a broth microdilution assay (EUVSEC, Sensititre; Trek Diagnostics, Ltd.), which includes the quinolones ciprofloxacin and nalidixic acid. Isolates were classified as resistant (R) if they grew on or above the ECOFF values for ciprofloxacin (R, >0.06 mg/liter) and/or nalidixic acid (R, >16 mg/liter) as defined by EUCAST (ECOFF values as of 8 January 2019). In addition, all isolates were tested for susceptibility to the following substances: tetracycline, ampicillin, sulfamethoxazole, trimethoprim, chloramphenicol, cefotaxime, ceftazidime, gentamicin, azithromycin, meropenem, colistin, and tigecycline. Azithromycin was excluded from further data analyses, as no ECOFF has as yet been defined for this compound. In the present study, QREC isolates from two livestock species and two wild animal species, specifically broilers, pigs, wild birds, and red foxes, were included. Broiler and pig isolates were chosen due to their relatively high number of samples positive for QREC by selective screening compared to other Norwegian livestock species (47) as well as the number of available isolates. Isolates were grouped according to MIC values for ciprofloxacin and nalidixic acid and according to the total number of antimicrobial substances to which they were resistant based on the Sensititre EUVSEC panel, resulting in 86 groups (see Table S1 in the supplemental material). A random selection within each group was done, representing each animal species where available. This grouping ensured phenotypic diversity among the isolates. Year of isolation and geographical location data for each isolate were collected where available. The resulting data set was composed of 285 isolates, where 88 isolates were from broilers, 75 were from pigs, 70 were from wild birds, and 52 were from red foxes. The overall occurrence of antimicrobial resistance among the isolates and per animal species included in this study is available in Table S2.

### DNA extraction.

Isolates stored at −80°C were plated onto MacConkey agar with 0.06 mg/liter ciprofloxacin to confirm resistance. DNA was extracted from colonies on the plate with the QIAamp DNA minikit (Qiagen), according to the manufacturer’s instructions. The DNA concentration was determined by using the broad-range DNA Qubit assay (Qiagen), and DNA quality was assessed by using the NanoDrop One spectrophotometer (Thermo Scientific). A Fragment Analyser automated capillary electrophoresis system instrument (catalog number FSV2-DE2-100; Advanced Analytical) and gel electrophoresis were used to determine DNA integrity.

### Library preparation and sequencing.

Quality-controlled DNA (*n* = 212) was used for Nextera Flex (Illumina) library preparation and sequenced over two lanes in a HiSeq 3000 instrument (Illumina), spiked with PhiX for sequencing quality control, resulting in paired-end reads of 150 bp. The sequencing service was provided by the Norwegian Sequencing Centre (www.sequencing.uio.no/). The remaining isolates were previously sequenced at the same facility with Nextera XT library preparation on a HiSeq 2000 instrument (*n* = 29) or a HiSeq 2500 rapid-run instrument (*n* = 44), resulting in paired-end read lengths of 125 and 250 bp, respectively. For the last group, each sample was sequenced on two lanes, resulting in four fastq files per sample.

### Quality control and contaminant screening.

Sequences were quality controlled using FastQC (https://www.bioinformatics.babraham.ac.uk/projects/fastqc/) version 0.11.7. Potential contaminants were screened for by using Mash (48) version 1.1. A minimum identity value was set at 0.95. Bacterial species other than E. coli at levels above this threshold were deemed a significant contaminant. This excluded four isolates from all further analyses due to contamination with *Citrobacter* or *Enterobacter* reads. See Sections S3.2 and S3.3 in the supplemental material for results.

### Antimicrobial resistance gene identification and multilocus sequence typing.

In total, 19 different plasmid-mediated and chromosomal genes associated with quinolone resistance were investigated [chromosomal genes *gyrA*, *gyrB*, *parC*, *parE*, *marR*, *marA*, *soxR*, *robA*, and *rpoB* and plasmid-mediated genes *qnrA*, *qnrB*, *qnrC*, *qnrD*, *qnrS*, *qnrE*, *qnrVC*, *oqxAB*, *qepA*, and *aac(6′)-Ib-cr*]. The genes were selected based on their description in the literature as well as their presence in the antimicrobial resistance gene databases described below. Possible coselection of antimicrobial resistance was investigated by including all additional plasmid-mediated genes related to other antimicrobial resistance types in the database used.

The genes *gyrA*, *gyrB*, *parC*, and *parE* were screened for mutations in the QRDR (49). Specifically, the QRDR of GyrA is located between amino acids 67 and 106 (50). Based on alignments of QRDRs from another study (49) to those of E. coli K-12 versions of the genes, this region was in the other proteins defined to be between amino acids 333 and 481 for GyrB, between amino acids 51 and 170 for ParC, and between amino acids 366 and 523 for ParE. See Section S3.4 in the supplemental material for reference sequences. The remaining chromosomal genes were investigated for mutations in the whole gene. Only mutations that lead to amino acid substitutions, here called substitutions, were of interest. Only presence/absence was considered for plasmid-mediated genes. Phenotypic resistance patterns were compared to the genotype identified for each animal species.

Antimicrobial resistance gene detection and sequence type (ST) determination were done by analyzing raw reads with Antimicrobial Resistance Identification by Assembly (ARIBA) (51) version 2.12.1. The presence of plasmid-mediated genes was determined by comparison to the ResFinder (52) database (downloaded 4 September 2018), while mutations in chromosomal genes were determined by comparison to the MegaRes (53) database (downloaded 4 September 2018) (see Section S3.5 in the supplemental material for reference sequences). An R script was used to extract the above-mentioned genes from the ARIBA results (https://tinyurl.com/y3f35mj2). Flags reported by ARIBA were used to quality check the reported variant or gene (Section S3.6). Each novel substitution reported by ARIBA was verified by comparison to their subsequent assemblies.

STs were determined using the multilocus sequence typing (MLST) scheme hosted by EnteroBase (54). Isolates with STs that were not able to be identified were uploaded to EnteroBase for manual identification (https://enterobase.warwick.ac.uk/).

### Assembly, annotation, and core-gene analysis.

Residual PhiX was removed with BBduk version 38.20 (https://jgi.doe.gov/data-and-tools/bbtools/) by mapping k-mers to the PhiX genome (GenBank accession number NC_001422.1), using a k-mer size of 31. Trimmomatic (55) version 0.38 was subsequently used to trim adapter sequences and low-quality nucleotides using a minimum-length setting of 36 bp and a sliding window of 4:15, with the Trimmomatic NexteraPE-PE adapter file. SPAdes (56) version 3.12.0 was used to assemble genomes with the settings “careful” and “coverage cutoff auto.” Both the paired and singleton reads from Trimmomatic were used. Assembly error correction was performed with Pilon (57) version 1.22 by mapping the trimmed reads back to the assembly with BWA mem version 0.7.17 (http://bio-bwa.sourceforge.net/). Prokka (58) version 1.13 was utilized for gene annotation, with the genus setting as “*Escherichia*,” species setting as “*coli*,” and kingdom setting as “*Bacteria*.” Five complete E. coli reference genomes were downloaded from the National Center for Biotechnology Information (NCBI) database and used as annotation references (Table S3). Pangenome analysis was performed with Roary (59) version 3.12.0 using the MAFFT aligner. QUAST (60) version 4.6.3 was used to evaluate the assemblies (see Section S3.7 in the supplemental material for results). One isolate was excluded due to low assembly quality, in addition to the four above-mentioned isolates that were removed due to contamination. The final data set was thus composed of 280 isolates, of which 87 were from broilers, 75 were from pigs, 52 were from red foxes, and 66 were from wild birds.

### Phylogenetic analysis.

Snp-sites (61) version 2.4.1 was used to concatenate single nucleotide polymorphism (SNP) sites in the core gene alignment from Roary. The resulting SNP site alignment was used to reconstruct a maximum likelihood (ML) tree with IQTree (62) version 1.6.8. Branch supports were obtained using the ultrafast bootstrap approximation (UFBoot) (63) with 1,000 bootstrap replicates. ModelFinder (64) and ascertainment bias correction (ASC) (65) were used to determine the best-fitting evolutionary model. ASC was used to avoid branch length overestimation due to the absence of invariant sites in our data set. Annotation and tree visualization were done with ggtree (66). Snp-dists (https://github.com/tseemann/snp-dists) version 0.6.3 was used to identify the number of SNP differences between all isolates.

The phylogenetic tree was inspected to identify major clades with isolates showing low genetic divergence. To quantify the amount of genetic change, patristic distances were calculated from the total tree in R with the distTips function from the adephylo package (67). The patristic distance cutoff was set to 0.003 because it resulted in clades that predominantly contained isolates from a single ST (Fig. S1). Clades deemed of interest were selected based on the presence of isolates from different animal species, or from the same animal species but from different geographic locations, resulting in six clades.

New phylogenetic trees were created for each of the six clades by first aligning the pilon-corrected assemblies using ParSNP (68) version 1.2 to identify the core genome SNPs for the isolates in each clade. Harvesttools (68) version 1.2 was used for format conversion, followed by Gubbins (69) version 2.3.2 to screen for and remove possible recombinant sequences from the core SNP multifasta alignment using the GTRGAMMA model with RAxML as the tree builder. IQTree was subsequently used to generate an ML tree from the filtered polymorphic-site alignment using UFBoot and ModelFinder with ASC. SNP distances were calculated from the filtered polymorphic-site alignment from Gubbins with Snp-dists. Additionally, the fraction of shared genomes for isolate pairs differing by <20 SNPs was calculated with ParSNP. Isolates sharing >90% of their genomes were regarded as clones and were further investigated to uncover possible dissemination.

### Statistical analyses.

Statistical data, figures, and tables were generated with R version 3.6.1 (70).

Significances of differences between the observed and expected occurrences of resistance mechanisms among the four animal species were determined by χ^2^ tests. Correlations between the presences of specific genes were calculated using a Pearson correlation test, with a significance level of 0.05.

Basic summary statistics were calculated for the SNP distances for isolates within each animal species and for isolates within the selected clades. To determine whether isolates from one animal species clustered more closely than isolates from the other animal species, the median minimum pairwise SNP distance for isolates belonging to the same animal species was calculated. To evaluate if isolates belonging to each host species were more aggregated in the tree, i.e., had a shorter distance to another isolate from the same species than randomly expected, we performed a randomization test with 1,000 permutations. The median minimum pairwise SNP distance for isolates belonging to the same animal species was calculated for each iteration. *P* values were calculated on the basis of how many expected values from *x* iterations were below the observed values.

Nonmetric multidimensional scaling (NMDS) was used to identify the distribution of quinolone resistance mechanisms within each major ST cluster based on the presence (1) and absence (0) of quinolone-resistance-conferring substitutions and genes. Only isolates from the dominant STs were included (*n* > 9). Distances were calculated from the presence/absence data with the “dist” function using the method “binary.” The NMDS analysis was performed with the “metaMDS” function from the vegan package (71), with 200 random starts. A stress plot was calculated to determine how well the ordination represented the data (Fig. S2).

### Data availability.

Raw reads have been uploaded to the ENA database under BioProject accession numbers PRJEB36302, PRJEB33048, and PRJEB33043.

## Supplementary Material

Supplemental file 1
