# Supplementary material for: Dissemination of Quinolone-Resistant Escherichia coli in the Norwegian Broiler and Pig Production Chains and Possible Persistence in the Broiler Production Environment
Source: Appl Environ Microbiol. 2020 Mar 18;86(7):e02769-19. doi: 10.1128/AEM.02769-19 (PMC7082582; doi:10.1128/AEM.02769-19)
Supplement: Supplemental file 1 [file AEM.02769-19-s0001.pdf]

Supplementary material

Contents

Section 1: Supplementary tables **2**

Table S1: Group listing 2

Table S2: Occurrence of antimicrobial resistance among the selected quinolone resistant isolates. . 4

Table S3: Prokka reference genomes 5

Table S4: Identified amino acid substitutions in the QRDR of GyrA, ParC and ParE. 6

Table S5: QRDR substitutions in GyrA, ParC and ParE per animal species. 7

Table S6: AA substitutions in MarA, MarR, RobA, RpoB and SoxR. 9

Table S7: Mechanisms in isolates without substitutions in QRDR 10

Table S8: Other plasmid mediated genes 11

Table S9: Co-resistance 12

Table S10: SNP distance statistics on isolates from the same species. 13

Section 2: Supplementary figures **14**

Figure S1: Clade selection based on patristic distances 14

Figure S2: Stressplot 15

Figure S3: Correlations between chromosomal genes and plasmid mediated genes 16

Figure S4: Iteration analysis results 17

Figure S5: Maximum likelihood tree, Clade A 18

Figure S6: Maximum likelihood tree, Clade C 19

Figure S7: Maximum likelihood tree, Clade D 20

Figure S8: Maximum likelihood tree, Clade E 21

Section 3: Metadata and reference sequences **22**

3.1: Isolate information and metadata 22

3.2: FastQC analysis results 22

3.3: Mash screen results 22

3.4: QRDR determination of GyrB, ParC and ParE 22

3.5: ARIBA flag selection 22

3.6: MEGARes and ResFinder reference sequences 22

3.7: QUAST results 23

References **24**

Section 1: Supplementary tables

Table S1: Group listing

The table presents the different phenotypic groups used in the isolate selection, and the number of isolates from each animal species in each group. The number of resistances within each group is listed in the column "No. of resistances".

Group	No. of resistances	CIP	NAL	Isolates per species	Total isolates
1	1	0.06	32	Pig (3), Wild bird (1), Red fox (2)	6
2	1	0.06	64	Red fox (1)	1
3	1	0.25	4	Pig (1), Broiler (1)	2
4	1	0.25	8	Pig (3), Wild bird (1)	4
5	1	0.25	16	Pig (4)	4
6	1	0.5	4	Pig (1)	1
7	1	0.5	8	Wild bird (2)	2
8	1	0.5	16	Pig (2), Wild bird (3)	5
9	2	0.06	32	Pig (1)	1
10	2	0.12	32	Broiler (4), Wild bird (1)	5
11	2	0.12	64	Broiler (1), Wild bird (1), Red fox (1)	3
12	2	0.12	128	Broiler (5), Wild bird (1)	6
13	2	0.25	8	Pig (1), Wild bird (1), Red fox (2)	4
14	2	0.25	32	Broiler (1)	1
15	2	0.25	64	Pig (2), Broiler (5)	7
16	2	0.25	128	Broiler (11), Pig (2), Wild bird (1), Red fox (3)	17
17	2	0.25	256	Broiler (1), Pig (4), Wild bird (4), Red fox (3)	12
18	2	0.5	8	Pig (1)	1
19	2	0.5	16	Wild bird (1)	1
20	2	0.5	32	Pig (1)	1
21	2	0.5	128	Broiler (2)	2
22	2	0.5	256	Broiler (3)	3
23	2	1	256	Broiler (1), Wild bird (1), Red fox (2)	4
24	2	4	256	Wild bird (1)	1
25	2	8	256	Red fox (1)	1
26	3	0.12	32	Broiler (2)	2
27	3	0.12	64	Red fox (3), Broiler (2)	5
28	3	0.12	128	Broiler (3), Pig (1)	4
29	3	0.25	4	Wild bird (1)	1
30	3	0.25	8	Pig (1), Red fox (1)	2
31	3	0.25	32	Broiler (1)	1
32	3	0.25	64	Broiler (4), Pig (1)	5
33	3	0.25	128	Broiler (10), Pig (4), Wild bird (4), Red fox (1)	19
34	3	0.25	256	Broiler (3), Pig (1), Wild bird (4), Red fox (5)	13
35	3	0.5	8	Pig (1)	1
36	3	0.5	16	Pig (1), Red fox (3)	4
37	3	0.5	256	Pig (1)	1
38	3	1	64	Broiler (1)	1
39	3	2	128	Broiler (1)	1
40	3	16	256	Pig (1), Wild bird (2), Red fox (1)	4

(continued)

Group	No. of resistances	CIP	NAL	Isolates per species	Total isolates
41	4	0.03	64	Pig (1)	1
42	4	0.12	64	Pig (1)	1
43	4	0.12	128	Broiler (2), Wild bird (2), Red fox (1)	5
44	4	0.12	256	Wild bird (1)	1
45	4	0.25	4	Wild bird (1), Red fox (3)	4
46	4	0.25	8	Wild bird (4)	4
47	4	0.25	16	Pig (1), Red fox (1)	2
48	4	0.25	128	Broiler (3), Pig (2)	5
49	4	0.25	256	Broiler (1), Pig (2), Wild bird (3), Red fox (1)	7
50	4	0.5	16	Wild bird (2)	2
51	4	0.5	128	Pig (1)	1
52	4	0.5	256	Broiler (3), Pig (3), Wild bird (3)	9
53	4	1	256	Red fox (1)	1
54	4	4	256	Broiler (1)	1
55	4	8	256	Broiler (1)	1
56	5	0.12	128	Pig (2), Red fox (1)	3
57	5	0.12	256	Broiler (1)	1
58	5	0.25	4	Red fox (1)	1
59	5	0.25	64	Broiler (1), Red fox (2)	3
60	5	0.25	128	Broiler (2), Pig (4), Wild bird (1)	7
61	5	0.25	256	Pig (1), Red fox (1)	2
62	5	0.5	16	Pig (1), Wild bird (4)	5
63	5	0.5	64	Broiler (1)	1
64	5	0.5	128	Broiler (1), Red fox (1)	2
65	5	0.5	256	Broiler (1)	1
66	5	1	64	Broiler (1)	1
67	5	4	256	Pig (1)	1
68	5	16	256	Pig (1), Wild bird (3), Red fox (1)	5
69	6	0.12	32	Pig (1), Wild bird (1)	2
70	6	0.12	64	Pig (2)	2
71	6	0.25	4	Pig (1)	1
72	6	0.25	64	Pig (1)	1
73	6	0.25	128	Broiler (1), Pig (1), Wild bird (2), Red fox (1)	5
74	6	0.25	256	Pig (4), Red fox (3)	7
75	6	0.5	16	Wild bird (1), Red fox (1)	2
76	6	1	128	Broiler (1)	1
77	6	1	256	Wild bird (1), Red fox (1)	2
78	6	8	256	Broiler (1), Pig (1), Wild bird (5)	7
79	6	16	256	Pig (1), Red fox (1)	2
80	7	0.25	128	Red fox (1)	1
81	7	8	256	Broiler (4), Wild bird (1), Red fox (1)	6
82	7	16	256	Wild bird (3)	3
83	8	0.25	64	Wild bird (1)	1
84	8	0.5	128	Pig (3)	3
85	8	8	256	Red fox (1)	1
86	8	16	256	Pig (1)	1

Table S2: Occurrence of antimicrobial resistance among the selected quinolone resistant isolates.

The table presents the percent (%) occurrence of antimicrobial resistance among the selected isolates (n = 280) identified through minimum inhibitory concentration values in the Norwegian monitoring programme from 2006 to 2017. The epidemiological cut off values used were defined by EUCAST. Azithromycin was excluded as no epidemiological cutoff value is currently available.

Substance	Broiler[a]	Pig[b]	Red fox[c]	Wild bird[d]	Total[e]
Ciprofloxacin	100.0	93.3	94.2	98.5	96.8
Nalidixic acid	100.0	74.7	76.9	68.2	81.4
Tetracycline	26.4	49.3	46.2	60.6	44.3
Ampicillin	36.8	40.0	51.9	51.5	43.9
Sulfamethoxazole	28.7	50.7	36.5	36.4	37.9
Trimethoprim	17.2	36.0	44.2	36.4	31.8
Chloramphenicol	5.8	10.7	11.5	16.7	10.7
Cefotaxime	4.6	2.7	5.8	9.1	5.4
Ceftazidime	3.4	2.7	5.8	5.8	5.4
Gentamicin	0.0	5.3	3.8	9.1	4.3
Meropenem	0.0	0.0	0.0	0.0	0.0
Colistin	0.0	0.0	0.0	0.0	0.0
Tigecycline	0.0	1.3	0.0	0.0	0.4

[a] 87 isolates
[b] 75 isolates
[c] 52 isolates
[d] 66 isolates
[e] 280 isolates

Table S3: Prokka reference genomes

The genomes listed below were used as references for the Prokka annotation, and downloaded with ncbi-genome-download (https://github.com/kblin/ncbi-genome-download) with the following commands:

–refseq-category reference

–assembly-level complete

–genus “Escherichia coli” bacteria

Accession number	Information
GCF_000005845.2_ASM584v2	*E. coli* K12
GCF_000008865.2_ASM886v2	*E. coli* O157:H7 Sakai
GCF_000026345.1_ASM2634v1	*E. coli* IAI39
GCF_000183345.1_ASM18334v1	*E. coli* O83:H1 NRG 857C
GCF_000299455.1_ASM29945v1	*E. coli* O104:H4 2011C-3493

Table S4: Identified amino acid substitutions in the QRDR of GyrA, ParC and ParE.

The table presents the different amino acid substitutions identified in GyrA, ParC and ParE and percent occurrence in total for each amino acid substitution.

AA substitution	n	Total	Percent
**GyrA**			
S83L	170	280	60.7
None	51	280	18.2
S83L, D87N	33	280	11.8
D87Y	11	280	3.9
S83A	9	280	3.2
D87N	3	280	1.1
D87G	2	280	0.7
D87H	1	280	0.4
**GyrB**			
None	280	280	100.0
**ParC**			
None	237	280	84.6
S80I	28	280	10.0
A56T, S80I	8	280	2.9
S57T	2	280	0.7
S58I	2	280	0.7
S80R	2	280	0.7
S80I, E84V	1	280	0.4
**ParE**			
None	251	280	89.6
D475E	15	280	5.4
S458A	10	280	3.6
D463N	1	280	0.4
H516Y	1	280	0.4
L416F	1	280	0.4
L488M, A512T	1	280	0.4

Table S5: QRDR substitutions in GyrA, ParC and ParE per animal species.

The table presents each identified amino acid substitution in GyrA, ParC and ParE and their occurrence in isolates from each animal species. The percentage is based on the number of isolates per species with the mutation in question (n) in relation to the total number of isolates per animal species (Total).

Protein	AA substitution	n	Total	Percent
**Broiler**				
GyrA	S83L	75	87	86.2
GyrA	S83L, D87N	7	87	8.0
GyrA	D87N	2	87	2.3
GyrA	D87Y	2	87	2.3
GyrA	D87H	1	87	1.1
GyrB	None	87	87	100.0
ParC	None	79	87	90.8
ParC	S80I	8	87	9.2
ParE	None	73	87	83.9
ParE	D475E	13	87	14.9
ParE	L488M, A512T	1	87	1.1
**Pig**				
GyrA	S83L	45	75	60.0
GyrA	None	19	75	25.3
GyrA	S83L, D87N	5	75	6.7
GyrA	S83A	4	75	5.3
GyrA	D87Y	2	75	2.7
GyrB	None	75	75	100.0
ParC	None	66	75	88.0
ParC	A56T, S80I	4	75	5.3
ParC	S57T	2	75	2.7
ParC	S80R	2	75	2.7
ParC	S80I	1	75	1.3
ParE	None	70	75	93.3
ParE	S458A	3	75	4.0
ParE	D463N	1	75	1.3
ParE	L416F	1	75	1.3
**Red fox**				
GyrA	S83L	31	52	59.6
GyrA	None	10	52	19.2
GyrA	S83L, D87N	6	52	11.5
GyrA	D87G	2	52	3.8
GyrA	S83A	2	52	3.8
GyrA	D87Y	1	52	1.9
GyrB	None	52	52	100.0
ParC	None	42	52	80.8
ParC	S80I	8	52	15.4
ParC	A56T, S80I	1	52	1.9
ParC	S80I, E84V	1	52	1.9
ParE	None	49	52	94.2
ParE	D475E	2	52	3.8
ParE	S458A	1	52	1.9
**Wild bird**				

(continued)

Protein	AA substitution	n	Total	Percent
GyrA	None	22	66	33.3
GyrA	S83L	19	66	28.8
GyrA	S83L, D87N	15	66	22.7
GyrA	D87Y	6	66	9.1
GyrA	S83A	3	66	4.5
GyrA	D87N	1	66	1.5
GyrB	None	66	66	100.0
ParC	None	50	66	75.8
ParC	S80I	11	66	16.7
ParC	A56T, S80I	3	66	4.5
ParC	S58I	2	66	3.0
ParE	None	59	66	89.4
ParE	S458A	6	66	9.1
ParE	H516Y	1	66	1.5

Table S6: AA substitutions in MarA, MarR, RobA, RpoB and SoxR.

The table presents the identified amino acid substitutions in MarA, MarR, RobA, RpoB and SoxR. The column "n" denote the amount of isolates with the respective AA substitution. The percentage is relative to the total amount of isolates.

AA substitution	n	Total	Percent
**MarA**			
None	246	280	87.9
S127N	26	280	9.3
L78F	6	280	2.1
E33V	1	280	0.4
P76S	1	280	0.4
**MarR**			
G103S, Y137H	192	280	68.6
None	68	280	24.3
K62R, G103S, Y137H	6	280	2.1
S3N, G103S, Y137H	6	280	2.1
G103S, D118N, Y137H	3	280	1.1
A53E, G103S, Y137H	1	280	0.4
A53S, G103S, Y137H	1	280	0.4
E131K	1	280	0.4
G103S, D118N, H120K, Y137H	1	280	0.4
T102P, G103S, Y137H	1	280	0.4
**RobA**			
None	280	280	100.0
**RpoB**			
None	232	280	82.9
E320D	33	280	11.8
V261A	4	280	1.4
E320D, D485E	2	280	0.7
G1318S	2	280	0.7
D320E	1	280	0.4
E320D, D393E	1	280	0.4
E320D, F464C	1	280	0.4
E412D, T595I	1	280	0.4
H165Y	1	280	0.4
P847S	1	280	0.4
V980L	1	280	0.4
**SoxR**			
None	209	280	74.6
T38S, G74R	33	280	11.8
G74R	26	280	9.3
I40V	4	280	1.4
A111T	3	280	1.1
T38S, N45D, G74R	3	280	1.1
A24T, G74R	1	280	0.4
E115K	1	280	0.4

Table S7: Mechanisms in isolates without substitutions in QRDR

This table presents the mechanisms identified in isolates with no amino acid substitutions in either of GyrA, GyrB, ParC and/or ParE.

marA	marR	robA	rpoB	soxR	PMQR	n
0	0	0	0	0	qnrB19	12
0	0	0	0	0	qnrS2	4
0	0	0	1	0	qnrS1	1
0	1	0	0	0	qnrA1	1
0	1	0	0	0	qnrB19	5
0	1	0	0	0	qnrS1	17
0	1	0	0	0	qnrS2	2
0	1	0	0	0	qnrS4	1
0	1	0	0	1	qnrB19	1
0	1	0	0	1	qnrS1	2
0	1	0	1	0	qnrB19	1
0	1	0	1	0	qnrS1	1
1	1	0	1	1	qnrB19	1

Table S8: Other plasmid mediated genes

The table presents the plasmid mediated genes related to other resistance phenotypes among the isolates.

Gene	Absent	Present	Total	Percent	Included
bla$_{\text{TEM-1B}}$	182	98	280	35.00	Yes
aph6Id	192	88	280	31.43	Yes
aph3Ib	194	86	280	30.71	Yes
tetA	202	78	280	27.86	Yes
sul2	207	73	280	26.07	Yes
tetB	231	49	280	17.50	Yes
sul1	253	27	280	9.64	Yes
aadA1	254	26	280	9.29	Yes
dfrA5	255	25	280	8.93	Yes
catA1	257	23	280	8.21	Yes
dfrA17	259	21	280	7.50	Yes
aadA5	260	20	280	7.14	Yes
dfrA1	261	19	280	6.79	Yes
dfrA14	263	17	280	6.07	Yes
aph3Ia	266	14	280	5.00	Yes
mphA	268	12	280	4.29	No
aac(3')-IId	270	10	280	3.57	Yes
aadA13	271	9	280	3.21	Yes
sul3	271	9	280	3.21	Yes
bla$_{\text{TEM-1C}}$	273	7	280	2.50	Yes
aadA2	275	5	280	1.79	Yes
dfrA12	275	5	280	1.79	Yes
floR	275	5	280	1.79	Yes
bla$_{\text{CMY-2}}$	277	3	280	1.07	Yes
bla$_{\text{CTX-M-15}}$	277	3	280	1.07	Yes
bla$_{\text{CTX-M-55}}$	277	3	280	1.07	Yes
cmlA1	277	3	280	1.07	Yes
mphB	277	3	280	1.07	No
aac(3')-IIa	278	2	280	0.71	Yes
aadA12	278	2	280	0.71	Yes
aadA22	278	2	280	0.71	Yes
bla$_{\text{CTX-M-1}}$	278	2	280	0.71	Yes
bla$_{\text{SHV-2}}$	278	2	280	0.71	Yes
bla$_{\text{TEM-1A}}$	278	2	280	0.71	Yes
dfrA8	278	2	280	0.71	Yes
mphE	278	2	280	0.71	No
msrE	278	2	280	0.71	No
bla$_{\text{CTX-M-32}}$	279	1	280	0.36	Yes
bla$_{\text{SHV-12}}$	279	1	280	0.36	Yes
ermB	279	1	280	0.36	No
lnuF	279	1	280	0.36	No
tetD	279	1	280	0.36	Yes

Table S9: Co-resistance

The table presents the number of isolates with each combination of plasmid mediated resistance genes, excluding combinations represented by only a single isolate.

Combination of genes	Number of isolates
**PMQR Negative**	
tet	18
sul	2
dfrA	10
bla_{TEM}	5
bla_{SHV}	2
bla_{CMY}	3
aph	5
sul, *tet*	2
bla_{TEM}, *tet*	3
aph, *tet*	4
aph, *sul*	4
aadA, *dfrA*	4
bla_{TEM}, *sul*, *tet*	2
aph, *sul*, *tet*	2
aph, *dfrA*, *sul*	4
aph, bla_{TEM}, *tet*	6
aph, bla_{TEM}, *sul*	2
aadA, bla_{TEM}, *sul*	3
aph, bla_{TEM}, *sul*, *tet*	2
aph, bla_{TEM}, *dfrA*, *sul*	9
aph, $bla_{\mathrm{CTX\text{-}M}}$, *sul*, *tet*	2
aadA, *dfrA*, *sul*, *tet*	2
aadA, bla_{TEM}, *sul*, *tet*	2
aadA, bla_{TEM}, *dfrA*, *tet*	2
aph, bla_{TEM}, *dfrA*, *sul*, *tet*	10
aadA, bla_{TEM}, *dfrA*, *sul*, *tet*	2
aac(3')-II, *aph*, bla_{TEM}, *sul*, *tet*	2
catA, *aadA*, *dfrA*, *sul*, *tet*	2
catA, *aadA*, bla_{TEM}, *dfrA*, *tet*	2
aadA, *aph*, bla_{TEM}, *dfrA*, *sul*, *tet*	9
catA, *aadA*, *aph*, bla_{TEM}, *dfrA*, *sul*, *tet*	8
catA, *aac(3')-II*, *aadA*, bla_{TEM}, *dfrA*, *sul*, *tet*	2
catA, *aac(3')-II*, *aadA*, *aph*, bla_{TEM}, *dfrA*, *sul*, *tet*	2
**PMQR Positive**	
qnr	14
qnr, *tet*	2
bla_{TEM}, *qnr*	4
aph, *qnr*	3
bla_{TEM}, *qnr*, *tet*	5
bla_{TEM}, *dfrA*, *qnr*, *tet*	4
aph, bla_{TEM}, *qnr*, *tet*	2
aac(3')-II, $bla_{\mathrm{CTX\text{-}M}}$, *qnr*, *tet*	3
aadA, *dfrA*, *qnr*, *sul*, *tet*	3
aph, bla_{TEM}, *dfrA*, *qnr*, *sul*, *tet*	2

Table S10: SNP distance statistics on isolates from the same species.

The table presents the summary statistics on the minimum SNP distance to the closest isolate from same animal species.

Statistic	Broiler	Pig	Red fox	Wild bird
Min	0	0	0	0
1st.Q	2	2	9	1
Median	12	212	1388	53
Mean	3193	4310	7210	5323
3rd.Q	52	3471	12945	7922
Max	75534	47826	46955	33629

Section 2: Supplementary figures

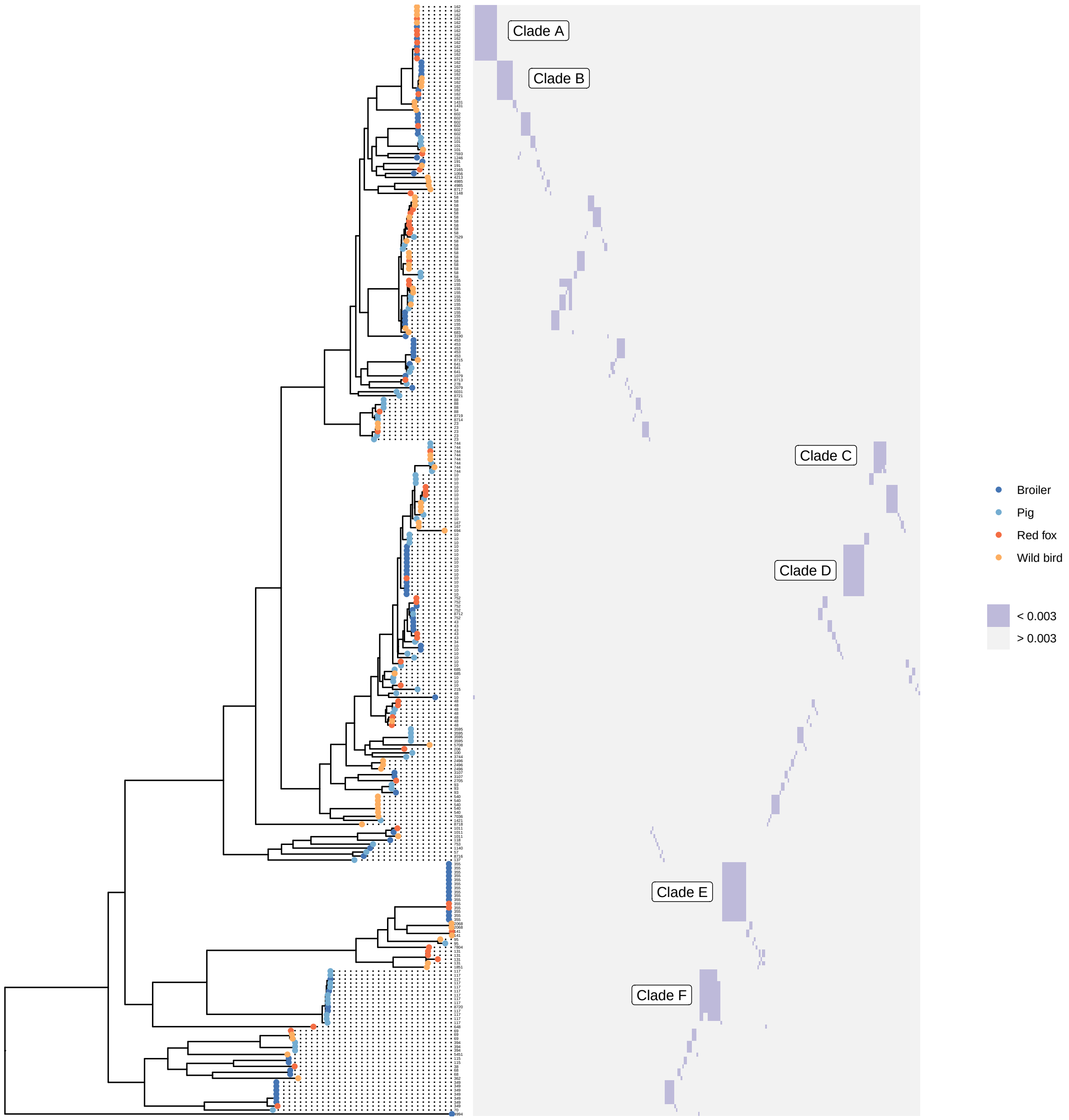

Figure S1: Clade selection based on patristic distances

The figure presents the overall phylogenetic tree (left) and the calculated patristic distances (right) as less than (<) or higher than (>) the specified cutoff value (0.003). The denoted clades were further investigated with phylogenetic analyses. Patristic distances were calculated from the core gene SNP tree.

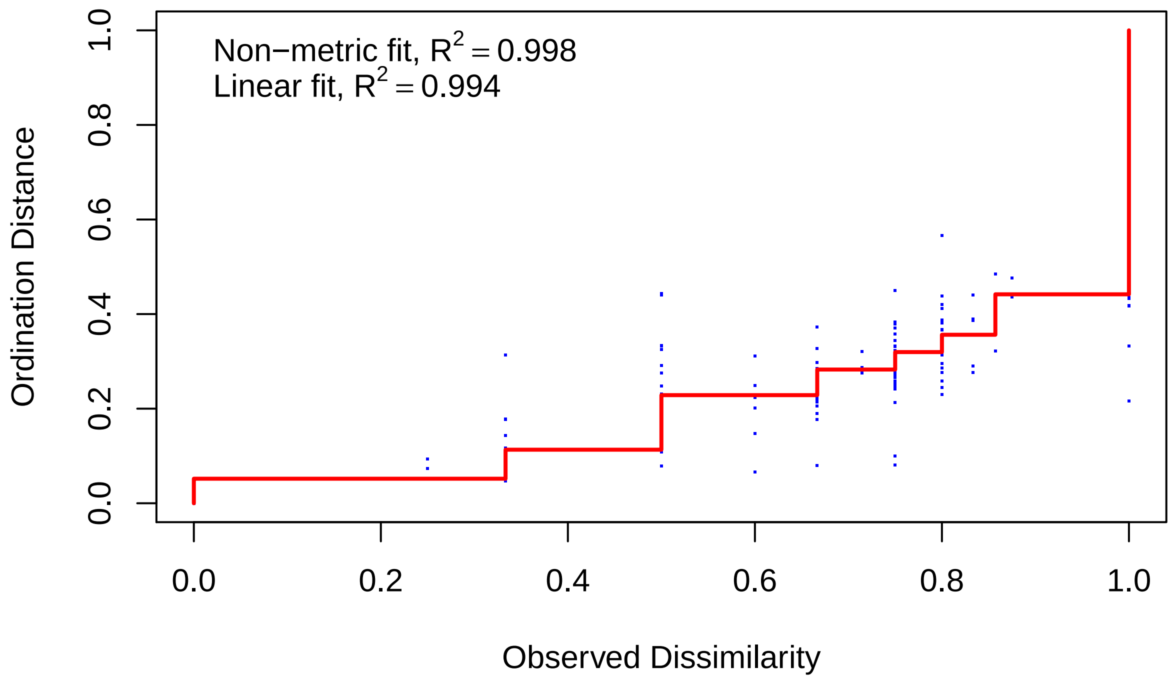

Figure S2: Stressplot

Non-metric multidimensional scaling (NMDS) was used to see if the distribution of quinolone resistance mechanisms from some sequence types were more homogenous than in other sequence types. The stressplot visualizes the goodness of fit.

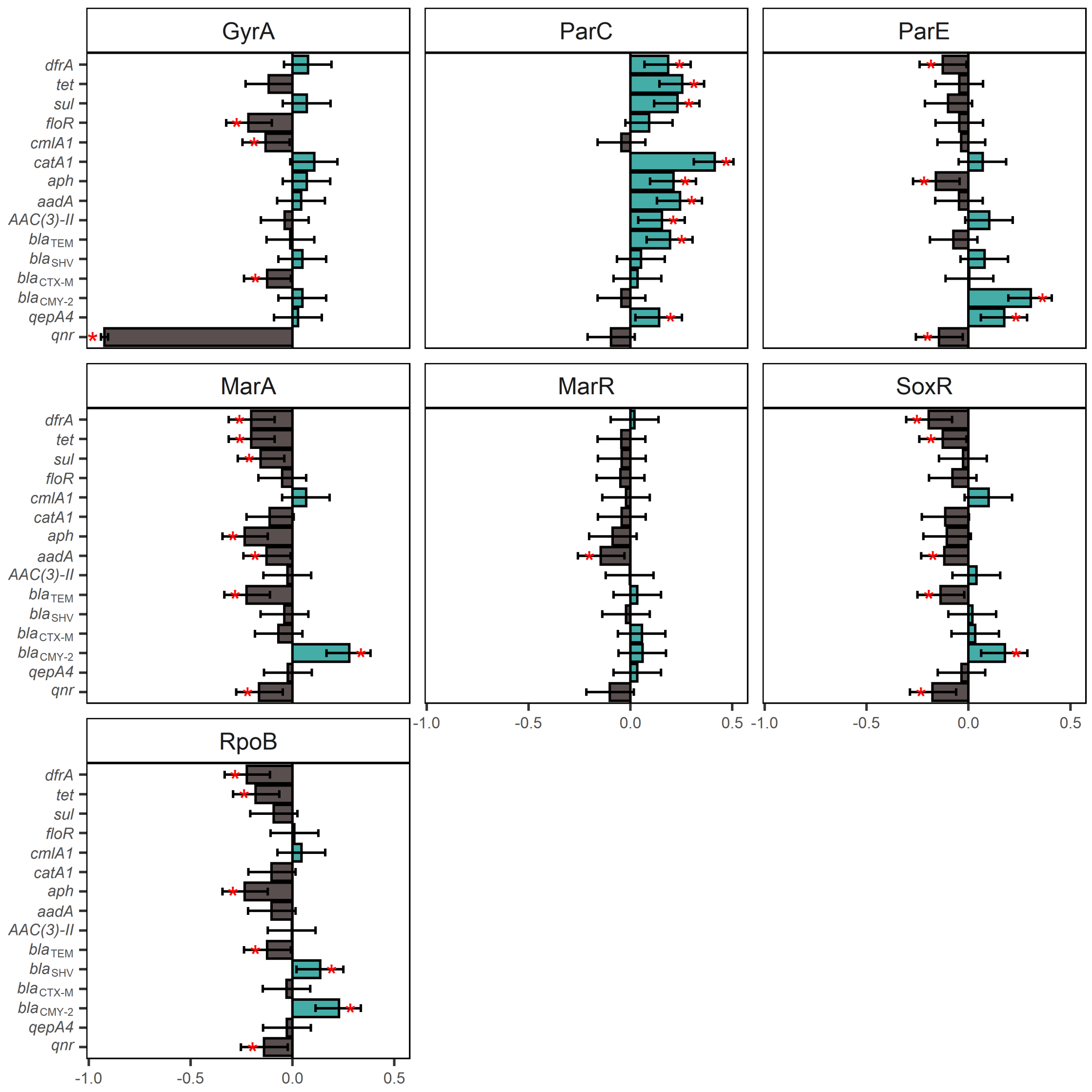

Figure S3: Correlations between chromosomal genes and plasmid mediated genes

The figure presents the Pearson correlation values for amino acid substitutions in GyrA, ParC, ParE, MarA, MarR, RpoB, and SoxR against all plasmid mediated genes identified. Grey = negative correlation, green = positive correlation. Black lines denote 95% confidence intervals. Red stars denote significant correlations. The plasmid mediated genes are grouped based on gene family: *dfrA* = *dfrA1*, *dfrA5*, *dfrA8*, *dfrA12*, *dfrA14*, and *dfrA17*. *tet* = *tetA*, *tetB*, and *tetD*. *sul* = *sul1 - 3*. *aph* = *aph3Ia*, *aph3Ib*, and *aph6Id*. *aadA* = *aadA1*, *aadA2*, *aadA5*, *aadA12*, *aadA13*, and *aadA22*. *AAC(3')-II* = *AAC(3')-IIa* and *AAC(3')-IId*. bla_{TEM} = $bla_{\text{TEM-1-A}}$ – $bla_{\text{TEM-1-C}}$. bla_{SHV} = $bla_{\text{SHV-2}}$ and $bla_{\text{SHV-12}}$. $bla_{\text{CTX-M}}$ = $bla_{\text{CTX-M-1}}$, $bla_{\text{CTX-M-15}}$, $bla_{\text{CTX-M-32}}$, and $bla_{\text{CTX-M-55}}$. *qnr* = *qnrA1*, *qnrB19*, *qnrS1*, *qnrS2*, and *qnrS4*.

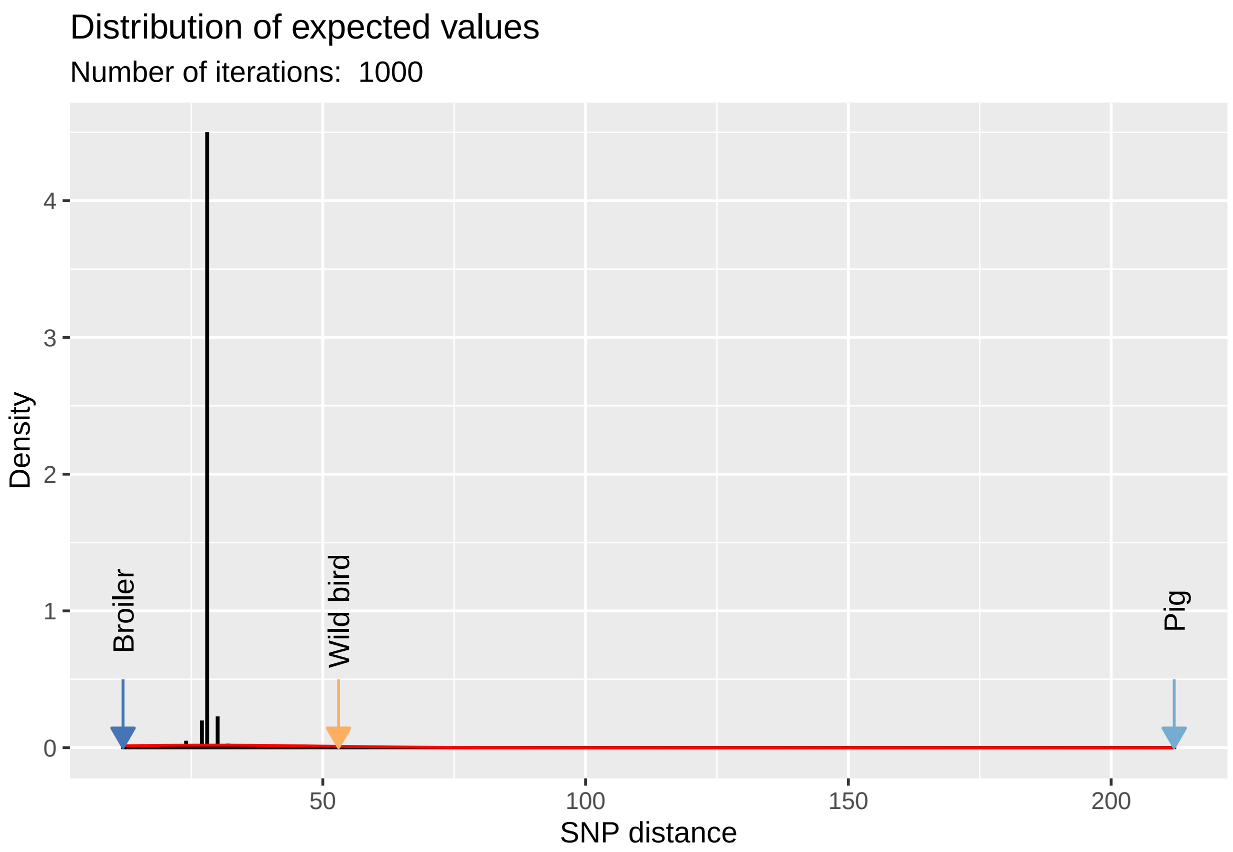

Figure S4: Iteration analysis results

The figure presents the observed median minimum SNP distance values for each animal species (coloured arrows) in relation to the expected values from the iteration analysis. Red fox results is excluded from the plot as the observed distance values were too high to visualize. The figure shows that isolates from broilers are more closely aggregated in the phylogenetic tree than what is randomly expected.

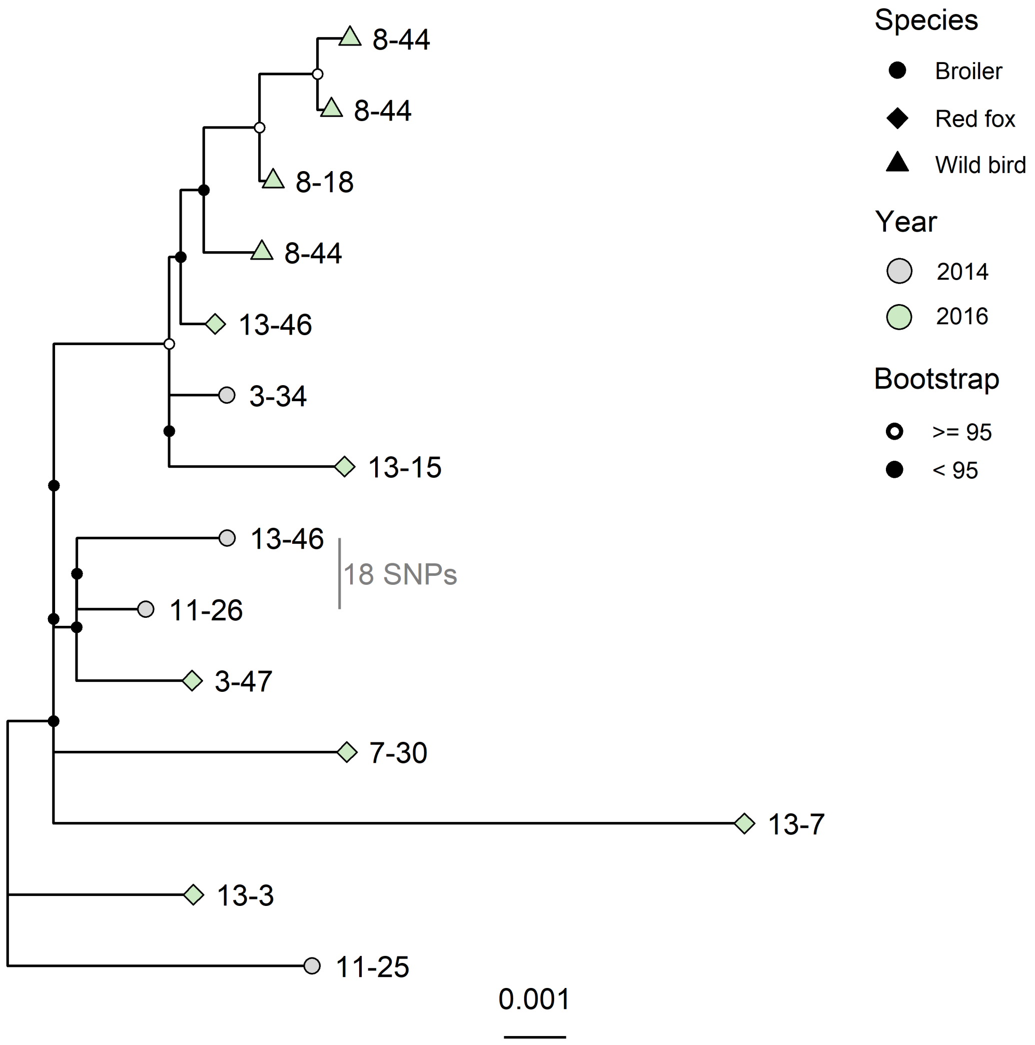

Figure S5: Maximum likelihood tree, Clade A

The values on each node is the UF Bootstrap values calculated by IQTree. The tip point shape represent the animal species of origin, and the color the year of isolation. The tip labels represent the location of the isolate; the first number represent the county, and the second number the municipality. Evolutionary model: K2P+ASC+R2. Shared genome: 88.4%.

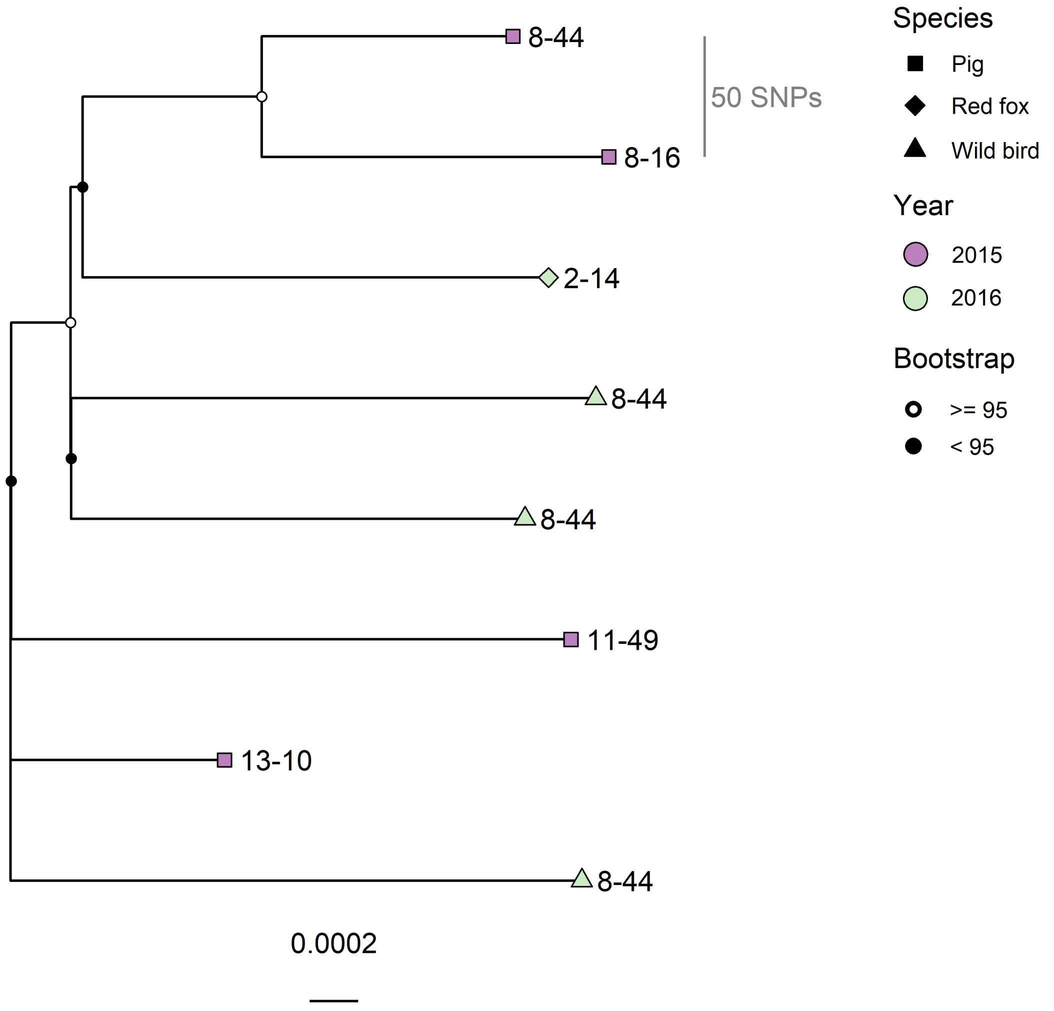

Figure S6: Maximum likelihood tree, Clade C

The values on each node is the UF Bootstrap values calculated by IQTree. The tip point shape represent the animal species of origin, and the color the year of isolation. The tip labels represent the location of the isolate; the first number represent the county, and the second number the municipality. Evolutionary model: K3P+ASC. Shared genome: 88.4%.

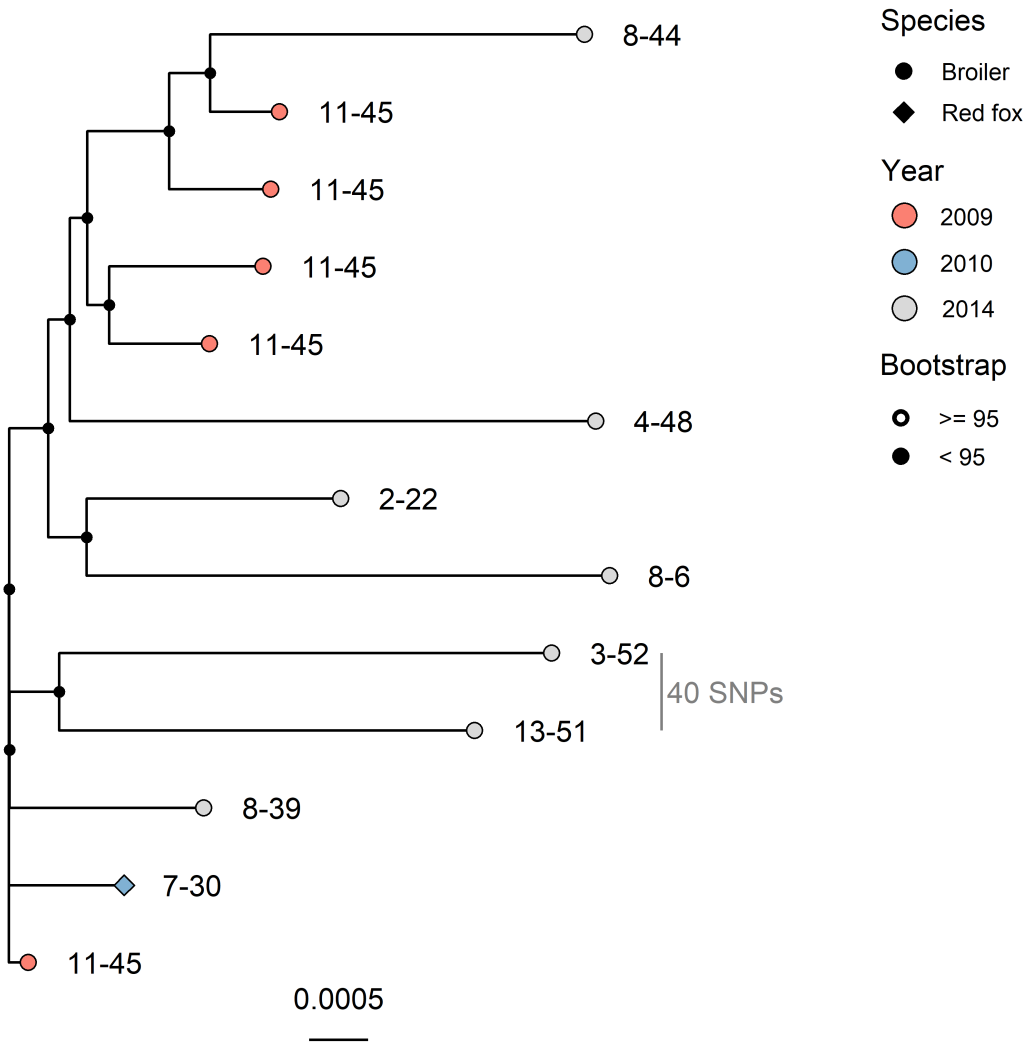

Figure S7: Maximum likelihood tree, Clade D

The values on each node is the UF Bootstrap values calculated by IQTree. The tip point shape represent the animal species of origin, and the color the year of isolation. The tip labels represent the location of the isolate; the first number represent the county, and the second number the municipality. Evolutionary model: K2P+ASC+R2. Shared genome: 87.2%.

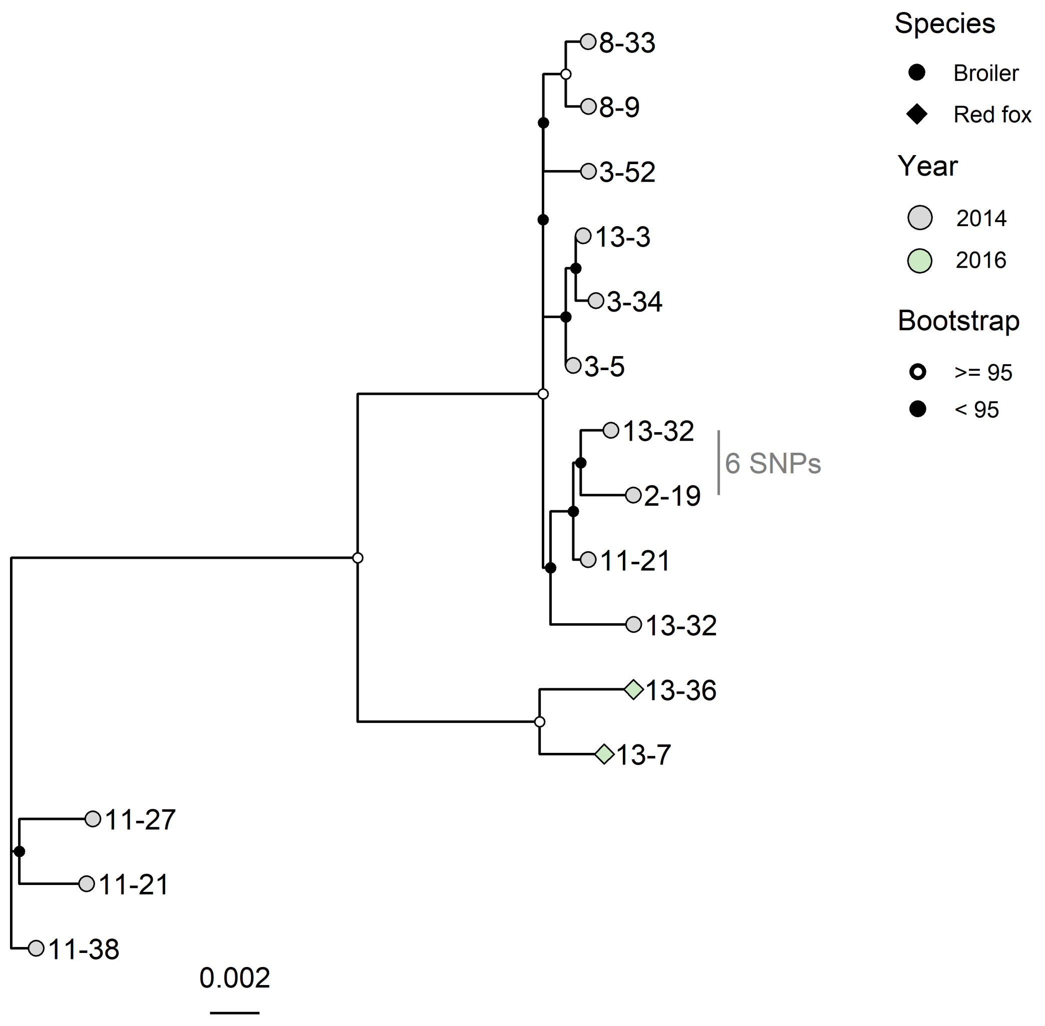

Figure S8: Maximum likelihood tree, Clade E

The values on each node is the UF Bootstrap values calculated by IQTree. The tip point shape represent the animal species of origin, and the color the year of isolation. The tip labels represent the location of the isolate; the first number represent the county, and the second number the municipality. Evolutionary model: K3P+ASC. Shared genome: 90.2%.

Section 3: Metadata and reference sequences

3.1: Isolate information and metadata

Metadata for all 280 included isolates can be downloaded here:

https://norwegianveterinaryinstitute.github.io/qm_wgs_4species/total_data_new.xlsx

3.2: FastQC analysis results

Raw read quality control results can be accessed here:

https://norwegianveterinaryinstitute.github.io/qm_wgs_4species/notebooks/fastqc_analysis.html

3.3: Mash screen results

Contaminant screening results can be accessed here:

https://norwegianveterinaryinstitute.github.io/qm_wgs_4species/notebooks/mash_analysis.html

3.4: QRDR determination of GyrB, ParC and ParE

The K12 reference sequence on the whole protein is listed as the first entry in each file. The rest of the fasta entries are taken from Jiménez Gómez et al. 2004 [1].

GyrB:

https://norwegianveterinaryinstitute.github.io/qm_wgs_4species/reference_genes/gyrB_QRDR_ref.fasta

ParC:

https://norwegianveterinaryinstitute.github.io/qm_wgs_4species/reference_genes/parC_QRDR_ref.fasta

ParE:

https://norwegianveterinaryinstitute.github.io/qm_wgs_4species/reference_genes/parE_QRDR_ref.fasta

3.5: ARIBA flag selection

Data on ARIBA flag selection can be downloaded here:

https://norwegianveterinaryinstitute.github.io/qm_wgs_4species/ariba_flag_selection.xlsx

3.6: MEGARes and ResFinder reference sequences

MEGARes:

https://norwegianveterinaryinstitute.github.io/qm_wgs_4species/reference_genes/total_megares_references.fa

ResFinder:

https://norwegianveterinaryinstitute.github.io/qm_wgs_4species/reference_genes/total_resfinder_references.fa

3.7: QUAST results

Assembly metrics can be accessed here:

https://norwegianveterinaryinstitute.github.io/qm_wgs_4species/notebooks/assembly_metrics.html

References

[1] P.A. JG, J.E. G de los R, A. RM, P. de PR, R. GA. 2004. Molecular basis of quinolone resistance in Escherichia coli from wild birds. Canadian Journal of Veterinary Research 68:229–231
